# A Novel Polysaccharide Purified from *Tricholoma matsutake*: Structural Characterization and In Vitro Immunological Activity

**DOI:** 10.3390/foods14061031

**Published:** 2025-03-18

**Authors:** Shuangmin Liang, Qi Guo, Jun Li, Ping Zhao, Changrong Ge, Shijun Li, Zhichao Xiao

**Affiliations:** 1 Livestock Product Processing and Engineering Technology Research Center of Yunnan Province, Yunnan Agricultural University, Kunming 650201, China; liangsm061211@126.com (S.L.); m18288650551@163.com (Q.G.); gcrzal@126.com (C.G.); 2 College of Food Science and Technology, Yunnan Agricultural University, Kunming 650201, China; 3 College of Plant Protection, Yunnan Agricultural University, Kunming 650201, China; aqulj@126.com; 4 Yunnan Agricultural University, Kunming 650201, China; ynau130@163.com

**Keywords:** *Tricholoma matsutake* polysaccharide, separation and purification, structural characterization, immunological activity

## Abstract

*Tricholoma matsutake*, as a rare wild edible mushroom, is popular due to its unique flavor and taste, as well as high nutritional and economic value. Investigating the relationship between the complex structure and in vitro immunological activity of TMP-2a, a novel polysaccharide isolated from *T. matsutake*, was the aim of this study. The results showed that TMP-2a consisted of six monosaccharides, fucose, glucosamine hydrochloride, galactose, glucose, mannose, and glucuronic acid, with molar ratios of 8.8:0.6:23.4:48.1:15.1:4.0 and a molecular weight of 27,749 Da. Furthermore, TMP-2a was mainly composed of →6)-β-Glc*p*-(1→ with →3)-β-D-Glc*p*-(1→ forming the main chain, with a small amount of →2,6)-α-D-Man*p*-(1→ and →6)-α-D-Gal*p*-(1→ structural units attached, and the branched chain was mainly composed of β-Glc*p*-(1→ or a small amount of α-L-Fuc*p*-(1→ as a telosaccharide attached at the O-6 position of →3,6)-β-D-Glc*p*-(1→. TMP-2a enhanced the proliferation and phagocytic activity of mouse macrophage RAW264.7, as well as the secretion of NO and cytokines (TNF-α, IL-6, IL-1β) to a considerable degree, maybe attributable to its glucan structure and the elevated presence of (1→3)-β-D-Glc*p* glycosidic bonds. This study establishes a basis for the structural identification and comprehensive investigation of the functional activities of *T. matsutake* polysaccharides while also offering a theoretical framework for the creation of *T. matsutake*-related food products.

## 1. Introduction

*Tricholoma matsutake* is a form of fungus that is widely available in Asian countries including China, Japan, Korea, and others [1]. It is a very popular and expensive fungus, containing a variety of nutrients, such as polysaccharides, protein, minerals, vitamins, and low fat [2]. Recent research has shown that a principal active component of *T. matsutake* is water-soluble polysaccharides [3]. Many clinical investigations have demonstrated that the polysaccharides isolated from *T. matsutake* (TMP) exhibit antitumor, immune-promoting, hematological, and antioxidant activities, meaning that they have a range of possibilities in the areas of health food and medicine [4].

However, research on TMP had primarily focused on its chemical composition and biological activities up to this point, with numerous papers on the TMP extraction procedure. Yin et al. [5] compared five methods for TMP extraction, with the highest yield of 8.11% for TMP extraction by counter-current probe ultrasonic extraction. Chen et al. [6] used an ultrasound-assisted technique to extract crude TMP with an extraction rate of 8.06%. In the previous study of our group, the optimal extraction process parameters for TMP extraction by ultrasonic-assisted water extraction and alcohol precipitation were obtained: water bath time of 2.25 h, material–liquid ratio of 1:31 g/mL, extraction temperature of 83.5 °C, and yield of 8.67% [7]. By using this method, the extraction rate was increased, the time was shortened, and the operation was simple and low-cost. Therefore, this extraction method was applied to the present study to establish a systematic study for the determination of TMP from extraction, isolation and purification, structure identification, and bioactivity.

At present, studies have mostly concentrated on crude TMP or the extracted and isolated portion of the polysaccharide for further structural and biological activity investigations, according to the reviewed research literature on TMP. The correlation between the biological activities of TMP and its structural mechanisms of action remains ambiguous, as structural variations in TMP from diverse sources present challenges in its structural analysis, thereby hindering the advancement and application of TMP. Ding et al. [8] and Yang et al. [9] studied polysaccharides from Sichuan *T. matsutake*, but the structural analysis of the polysaccharides only provided a rough description of the overall structural composition of the polysaccharides and could not analyze the linkages and spatial conformation. Cheng et al. [10] conducted a structural characterization of TMP, but there were differences in the extraction methods and the degree of isolation and purification, so the composition of the polysaccharides measured was small, which might result in differences in the structural resolution of the TMP.

Reduced immunity is thought to increase vulnerability to bacterial and viral infections, presenting a considerable risk to human health [11]. The examination of the conformational connection of polysaccharides can clarify variations in immunological activity and aid in the structural modification and chemical synthesis of polysaccharides. This study involved the isolation, purification, and structural elucidation of TMP based on prior extraction methods. The investigation of the immunological mechanisms of TMP and the examination of the correlation between its structure and biological function offered significant theoretical insights for the subsequent evaluation of its biological activity.

## 2. Materials and Methods

### 2.1. Materials and Reagents

*T. matsutake* was bought in Yimen County, Yunnan Province, China. RAW264.7 cells were obtained from the cell bank of the Shanghai Institute of Cell Biology.

### 2.2. Preparation of Crude TMP

**Figure 1:**
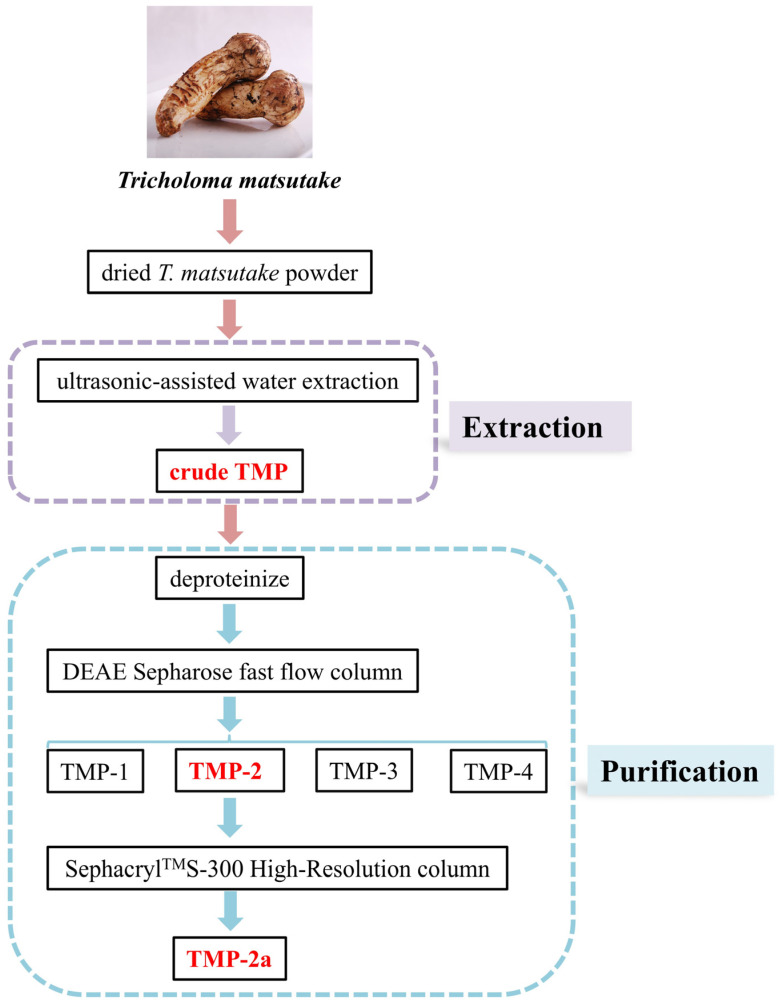
Flow diagram for the experimental extraction and purification of *T. matsutake* polysaccharides.

The dried *T. matsutake* slices were baked in an electric constant temperature blast drying oven (50 °C) for 24 h. The moisture content was reduced to 9.5% and then crushed and sieved to dried *T. matsutake* powder using a high-speed universal pulverizer. A total of 5.0 g of dried *T. matsutake* powder was weighed. The extraction temperature was 83.5 °C at an extraction material–liquid ratio of 1:31 g/mL. The solution was placed in an ultrasonic extractor (SCQ-9201B, Shanghai Shengyan Ultrasonic Instrument Co., Shanghai, China) for 20 min and in a water bath for 2.25 h. The solution was subsequently spun twice at 6500 rpm for 20 min. The supernatant was combined with anhydrous alcohol at a 1:3 ratio. The obtained solution was spun up for 15 min at 4000 rpm and stored at 4 °C for 24 h. The result was crude TMP. The preparation of crude TMP is shown in Figure 1.

### 2.3. Isolation and Purification of TMP

The crude TMP was deproteinized using the Sevage method. In total, 0.3 g of TMP was diluted in 40 mL of ultrapure water and subsequently passed through a DEAE Sepharose fast flow column (4.6 × 50 cm), eluting with distilled water and 0.1, 0.3, and 0.5 mol/L NaCl solutions to provide four fractions, designated TMP-1, TMP-2, TMP-3, and TMP-4. A total of 0.03 g of TMP-2 was purified using a Sephacryl^TM^S-300 High-Resolution column (2.6 × 90 cm) (CXZ, Shanghai Speed Tech Industrial Co., Shanghai, China) and flushed with a 0.1 mol/L NaCl solution. The same peak eluate was collected to obtain purified polysaccharide (TMP-2a). Isolation and purification of TMP are shown in Figure 1.

### 2.4. The Monosaccharide Composition of TMP-2a

The monosaccharide content was assessed with ion chromatography (IC) (ICS 5000, Thermo Fisher Scientific, Waltham, MA, USA). Sixteen monosaccharide standards—fucose (Fuc), rhamnose (Rha), arabinose (Ara), galactose (Gal), glucose (Glc), xylose (Xyl), mannose (Man), fructose (Fru), ribose (Rib), galacturonic acid (GalA), glucuronide (GlcA), aminogalactose hydrochloride (GalN), glucosamine hydrochloride (GlcN), N-acetyl-D-glucosamine (GlcNAc), guluronic acid (GulA), and mannuronic acid (ManA)—were formulated into standard solutions at an approximately 10 mg/mL concentration. The standard solutions of the monosaccharides were prepared as 0.1, 0.5, 1, 5, 10, 20, and 50 mg/L standard mixes. In total, 5.0 mg of the sample was weighed in an ampoule and 10 mL of 2 M trifluoroacetic acid (TFA) was added and hydrolyzed for 3 h at 120 °C. The solution was then blown dry with nitrogen and vortexed and mixed with 5 mL of distilled water. A total of 0.1 mL was pipetted into 0.9 mL of distilled water and centrifuged at 12,000 rpm for 5 min. The supernatant was taken into IC for analysis. The peak times of the mixed standards corresponded to the monosaccharide composition of the sample, respectively, and then the molecular weight and peak area of the standards were used to calculate the molar ratio of the monosaccharide composition [12]. The parameter conditions for the IC analysis were as follows: column: DionexCarbopac: DionexCarbopac^TM^PA20 (3 × 150); detector: electrochemical detector; mobile phase A: H_2_O; mobile phase B: 250 mM NaOH; mobile phase C: 50 mM NaOH and 500 mM NaAc; flow rate: 0.3 mL/min; inlet volume: 0.3 mL/min: flow rate: 0.3 mL/min; injection volume: 5 µL; column temperature: 30 °C. The mobile phase gradient system conditions were as follows: 0–20 min, A:B:C was 98.8:1.2:0; 20–30 min, A:B:C was 50.0:50.0:0; 30.1–46 min, A:B:C was 0:0:100.0; 46.1–50 min, A:B:C was 0:100:0; 50–80 min, A:B:C was 98.8:1.2:0.

### 2.5. The Molecular Weight Determination of TMP-2a

The molecular weight of polysaccharides was measured using high-performance gel permeation chromatography (HPGPC). A 5 mg/mL solution was made by weighing the samples and standards, separating them for 10 min at 12,000 rpm, filtering the supernatant, and then injecting it into an HPGPC system (LC-10A, Shimadzu, Kyoto, Japan). The parameters of the HPGPC analysis: column: BRT105-104-102 tandem gel column (8 × 300 mm); detector: OD detector (RI-502, Shodex, Tokyo, Japan); mobile phase: 0.05 M NaCl solution; flow rate: 0.6 mL/min; injection volume: 20 µL; column temperature: 40 °C.

### 2.6. The Fourier Transform Infrared Spectroscopy (FT-IR) Detection of TMP-2a

A total of 2.0 mg of TMP-2a and 200.0 mg of KBr were measured, combined, pulverized, and compressed into tablets. The FT-IR spectra were obtained within the frequency range of 4000 to 400 cm^−1^. The sections were analyzed within the region of 4000–400 cm^−1^ utilizing FT-IR 650 (Tianjin Gangdong Sci. & Tech. Development Co., Ltd., Tianjin, China) [13].

### 2.7. The Scanning Electron Microscope (SEM) Detection of TMP-2a

The morphological characteristics of TMP-2a were examined using SEM (Zeiss Sigma 300, Zeiss, Baden-Württemberg, Germany). TMP-2a was taken, adhered straight on the conductive adhesive, and then exposed to a 45 s gold spray utilizing a vacuum sputter coater set to 10 mA and 3 kV for the morphological images.

### 2.8. The Atomic Force Microscopy (AFM) Detection of TMP-2a

TMP-2a was solubilized in an aqueous solution and incrementally applied to the mica flakes, subsequently dried, and imaged using an AFM (Multimode 8, Bruker, Berlin, Germany).

### 2.9. The Particle Size and ζ-Potential Determination of TMP-2a

The particle size and ζ potential of TMP-2a were assessed utilizing a dynamic light scattering (DLS) particle size scanner (Zeta sizer NanoBrook Omni, Brookhaven Instruments Co., New York, NY, USA). TMP-2a was solubilized and dissolved in ultrapure water to a dosage level of 1 mg/mL. The particle size distribution was detected over a range of 0.3 nm–1.0 mm. The blank control utilized the refractive index and viscosity of water, measured at 1.33 and 0.89 cP, respectively.

### 2.10. The Methylation Detection of TMP-2a

Methylation can identify the linkage sites of monosaccharide residues and infer the proportion of monosaccharide residues in a polysaccharide molecule. In total, 2.0 mg of TMP-2a was mixed in 1 mL of anhydrous dimethyl sulfoxide, followed by the addition of 60.0 mg of NaOH. After 60 min of the reaction with the combination and CH_3_I at 30 °C in a water bath, 2 mL of ultrapure water was added to stop the methylation reaction. The mixture was hydrolyzed with 1 mL of 2 M TFA for 90 min, reduced with 60 mg of NaBH_4_ for 8 h at 25 °C, glacial acetic acid was added, and the mixture was dried at 101 °C. Subsequently, 1 mL of acetic anhydride was introduced, and the process was conducted at 100 °C for 1 h. The acetylated product was dispersed in chloroform to yield the derivative, which was analyzed by GC-MS (MS-QP 2010, Shimadzu, Kyoto, Japan). GC-MS analysis parameter conditions: column: RXI-5 SIL MS column (30 m × 0.25 mm × 0.25 μm); programmed heating conditions: starting temperature 120 °C, heating at 3 °C/min to 250 °C/min, holding for 5 min; inlet temperature: 250 °C; detector temperature: 250 °C/min; carrier gas: He; flow rate: 1 mL/min.

### 2.11. The Nuclear Magnetic Resonance (NMR) Detection of TMP-2a

A total of 50.0 mg of TMP-2a was solubilized in 0.5 mL of D2O and subsequently lyophilized. The sample was dissolved in 0.5 mL of D_2_O at 25 °C and determined by ^1^H NMR spectroscopy, ^13^C NMR spectroscopy, and DEPT135, COSY, HSQC, HMBC, and NOESY spectroscopy using a 600 MHz NMR spectrometer (Avance III HD, Bruker Corporation, Billerica, MA, USA). The mixing time for the NOESY experiment was 45.7 ms.

### 2.12. In Vitro Immunological Activity

#### 2.12.1. Cell Culture

RAW264.7 cells were cultivated in 5% CO_2_ at 37 °C in a high-glucose medium supplemented with 10% heat-inactivated FBS and 1% (*v*/*v*) penicillin–streptomycin antibiotics.

#### 2.12.2. Effect of TMP on Proliferation of RAW264.7 Cells

At a density of about 1 × 10^5^ cells, RAW264.7 cells were plated in 96-well plates and cultivated for 12 h. Various quantities of pure TMP (12.5, 25, 50, 100, 200 μg/mL) were introduced to the sample group, with each well containing a volume of 200 μL. LPS (10 μg/mL) was administered to the positive control group. After 48 h of continuous growth, 100 μL of MTT (0.5 mg/mL) was applied to each well. Subsequently, after 4 h of cultivation, 150 μL of DMSO was introduced, and the intensity of the absorption was quantified at 570 nm [14].

#### 2.12.3. Phagocytic Activity of TMP on RAW264.7 Cells

The planking and sample treatment of RAW264.7 cells were the same as the process described in 2.9.2. A total of 100 μL of a 0.075% (*w*/*v*) neutral red solution was added after 48 h. Following 1 h of incubation, 100 μL of cell lysate was incorporated and subsequently incubated overnight at 25 °C. The measurement wavelength was 540 nm [15].

#### 2.12.4. Effect of TMP on NO Release from RAW264.7 Cells

According to the the Griess kit (S0021S, Beyotime Biotechnology, Shanghai, China) operating instructions, the sample was added to the 96 well plate for processing. After 48 h, the supernatant was taken out and used for NO detection.

#### 2.12.5. Effect of TMP on Cytokines (TNF-α,IL-6,IL-1β) of RAW264.7 Cells

The specific operation was strictly in accordance with the ELISA kit (SP10205, SP10234, SP10180, Wuhan Saipei Biotechnology Co., Wuhan, China) operating instructions [16].

### 2.13. Statistical Analysis

The acquired data were statistically analyzed, and the difference was reported as the mean standard deviation. The data analysis utilized SPSS 19.0 statistical software, while graph production was performed with Origin 2018. *p* < 0.05 was deemed statistically significant, whereas *p* < 0.01 was regarded as highly significant.

## 3. Results and Discussion

### 3.1. Purification of TMP-2a by Sephacryl^TM^S-300 High-Resolution Column

**Figure 2:**
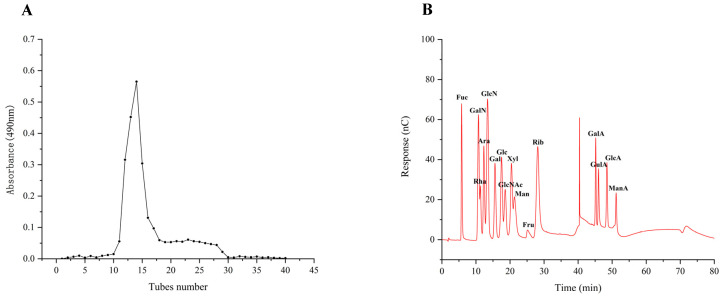

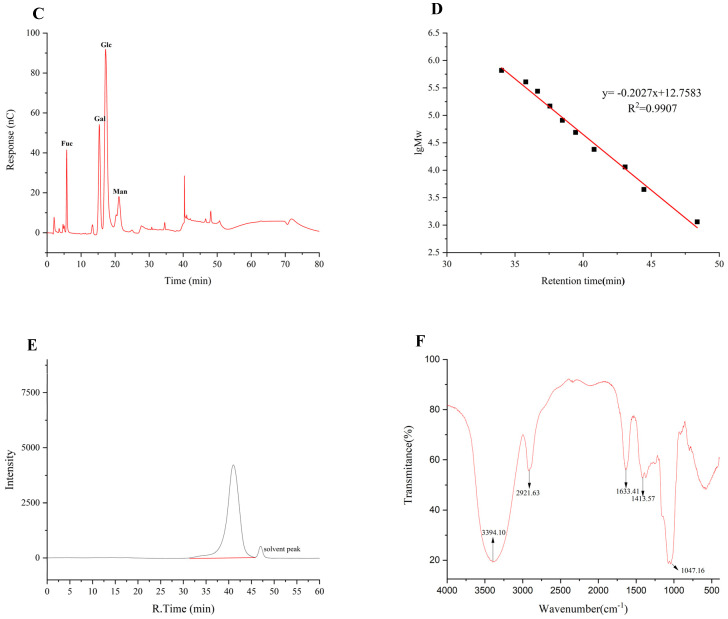
Fractionation pattern of TMP-2 by Sephacryl^TM^S-300 High-Resolution column (**A**); IC of 16 standard monosaccharides (**B**) and TMP-2a (**C**); standard curve of molecular weight (**D**); chromatogram of TMP-2a (**E**); FT-IR spectra of TMP-2a (**F**).

Prior research on the antioxidant and immunological properties of the four purified fractions revealed that TMP-2 was the most abundant fraction of TMP and exhibited superior antioxidant and immunological properties. Therefore, TMP-2 was selected to be filtered through the Sephacryl^TM^S-300 High-Resolution column (Figure 2A). TMP-2a had a production rate of 60.60 percent, a total sugar level of 97.97 percent, and a high purity.

### 3.2. The Analysis of Monosaccharide Composition of TMP-2a

The results of 16 mixed standard solutions of monosaccharides (Figure 2B) and TMP-2a (Figure 2C) were examined. The molar ratio of fucose, galactose, glucose, and mannose that made up TMP-2a was 8.1:22.6:56.8:12.5 (Table 1). Prior research has demonstrated that edible fungus possesses several heteropolysaccharides, abundant in glucose, fucose, galactose, mannose, and xylose. Polysaccharides with a similar monosaccharide composition of TMP-2a were also found in Lentinula edodes and Schizophyllum [17]. This study revealed that the monosaccharide composition of TMP-2a aligns with that reported by Cheng et al. [10], who identified TMP-5II as comprising glucose, galactose, mannose, and fucose in a molar ratio of 10.52:7.52:2.08:1.00. The percentage content of the monosaccharide composition was identical to that of TMP-2a, which was predominantly glucose. The polysaccharide breakdown and oligosaccharide exposure in this investigation were enhanced by the ultrasonic extraction method utilized for TMP [6].

### 3.3. The Determination of Molecular Weight of TMP-2a

Figure 2D displays the molecular weight standard curve. A major peak at 35–45 min can be seen in Figure 2E, indicating that TMP-2a was a homogeneous polysaccharide. The peak appearing at 46–48 min was the solvent peak (NaCl peak), which was also present in the studies of Wen et al. [18] and Li et al. [19]. The molecular weight of TMP-2a was 27,749 Da (Table 1). The ratio of the multiple dispersion coefficient heavy average molecular weight to the number average molecular weight, Mw/Mn, was 1.33, and the dispersion coefficient was close to 1, meaning that the molecular weight distribution was more homogeneous. TMP-2a was distributed at the 10,000 level, and the homogeneity of the molecular weight distribution was good. It differed from the homogeneous polysaccharide TMP-5II, which has a molecular weight of 15.76 kDa, as reported by Cheng et al. [10], and from TMP, which has a molecular weight of 72.14 kDa, as reported by Yang et al. [9]. Polysaccharides were chosen for their higher purity or content due to variations in the extraction and purification processes. Additionally, variations in the molecular weight and concentration of the monosaccharides affect the structural characterization and biological activity of polysaccharides, leading to polysaccharides exhibiting distinct biological activities.

### 3.4. The FT-IR Spectral Analysis of TMP-2a

Infrared spectroscopy is primarily employed for the identification of monosaccharide species, the analysis of glycosidic bond conformations in pyranose, the differentiation between furanose and pyranose forms, and the characterization of substituents on the sugar chain [20]. Figure 2F illustrates that the TMP-2a absorption band between 3600 and 3200 cm^−1^ corresponds to a stretching vibration absorption peak of -OH, with the absorption peaks in this range being characteristic of glycans. TMP-2a exhibited a distinct peak at about 3394 cm^−1^, confirming the existence of hydroxyl-OH in polysaccharides. The absorption peak at 2921 cm^−1^ was ascribed to the stretching vibration of C-H bonds, encompassing CH, CH_2_, and CH_3_ groups, which are characteristic peaks of carbohydrates. The absorption peak of TMP-2a was wider due to the presence of fucose. The absorption peak at 1633 cm^−1^ may represent the typical peak of water of crystallization. The absorption peak at approximately 1413 cm^−1^ corresponds to the C-O stretching vibration. The pronounced absorption peak at 1047 cm^−1^, located within the 1200–1000 cm^−1^ range, was ascribed to C-O-H and glycocyclic C-O-C, two variable angle vibration absorption peaks of the pyranoside ring backbone C-O, which are characteristic of pyranose rings, thereby indicating that TMP-2a comprises pyranose rings [21].

### 3.5. The Characterization of the Apparent Structure of TMP-2a

**Figure 3:**
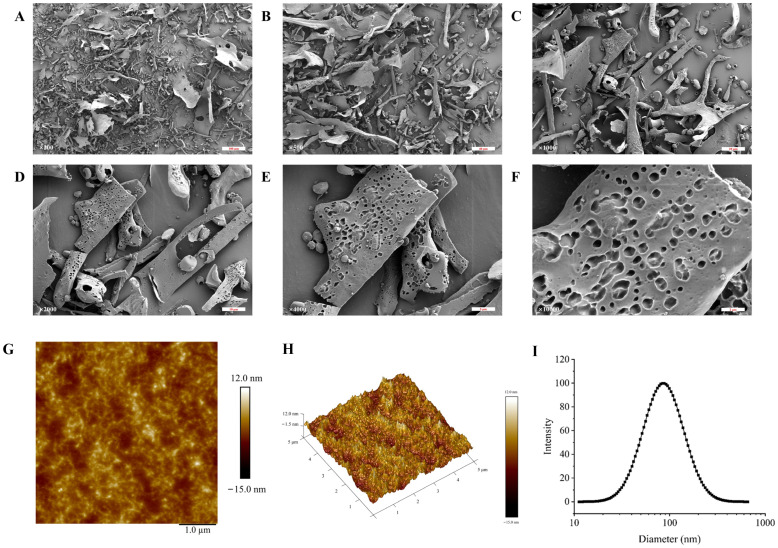
The characterization of the apparent structure of TMP-2a. SEM images at magnifications of 200×, 500×, 1000×, 2000×, 4000×, and 10,000× (**A**–**F**); AFM image (**G**) and 3D topography (**H**); The particle size distribution (**I**).

SEM is frequently used to describe a sample’s surface topography and microstructure. The SEM images of TMP-2a at different magnifications are shown in Figure 3A–F. Consistent with the findings of Tang et al. [22], the SEM observations revealed that TMP-2a comprised several flakes exhibiting diverse morphologies, alongside a few unevenly stacked rods. In addition, at 10,000× magnification, the surface of TMP-2a was rough with obvious pores, and this structure was similar to the extracellular polysaccharides produced by *Rhodococcus erythropolis* HX-2 studied by Hu et al. [23]. The porous structure was also more branched, and thus this type of porous structure of polysaccharides usually has higher viscosity and better water retention capacity, and a complex spatial structure and immunoreactivity [24].

The planar and stereo AMF images of TMP-2a are shown in Figure 3G,H. The planar AFM images showed a chain and irregular coil distribution of TMP-2a. AFM analysis showed that the height of TMP-2a was 7.3 nm. A single chain of natural polysaccharides usually had a diameter of 0.1 to 1.0 nm. The diameter of the sugar chain in TMP-2a was markedly larger than that of single-chain polysaccharides, suggesting the hypothesis of multiple sugar chain connections and entanglements, potentially due to van der Waals forces between polysaccharide molecules and hydrogen bonding among sugar chains [25].

The hydrodynamic characteristics of polysaccharides, including particle size distribution and potential, can indicate their stability to some degree. In general, smaller particle sizes of polysaccharide molecules and larger absolute values of the zeta potential facilitate the dispersion or dissolution of these molecules in a system. Conversely, bigger particle sizes and smaller zeta potential values promote aggregation [26]. TMP-2a aggregated to form particles in aqueous solution with a wide particle size distribution, as evidenced by Figure 3I, which shows that the particle size of TMP-2a in aqueous solution was 97.13 ± 0.64 nm and the Polymer Dispersity Index (PDI) value (0.24 ± 0.03) > 0.08. Table 1 illustrates that the zeta potential was −8.30 ± 0.45 mV, with an absolute value below 30, signifying that the TMP-2a dispersion system in aqueous solution was unstable and susceptible to aggregation.

### 3.6. The Methylation Analysis of TMP-2a

**Figure 4:**
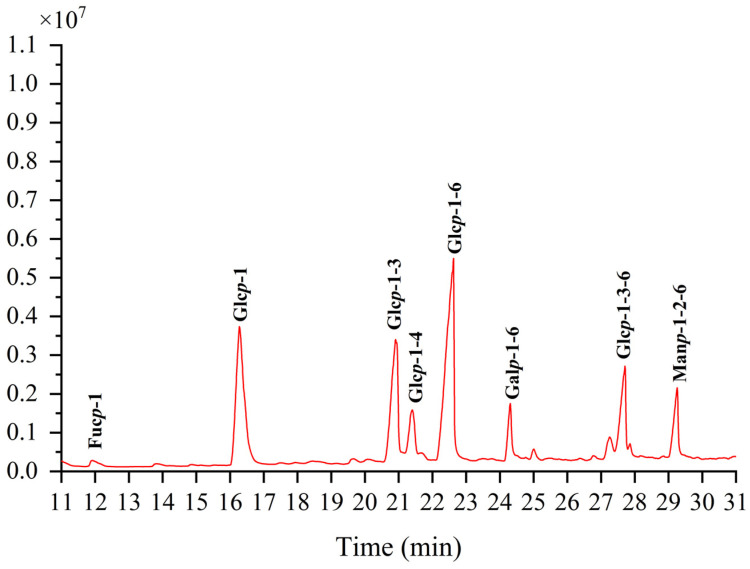
Ion chromatograms of GC-MS of TMP-2a.

Methylation can dictate the configuration of monosaccharides, the location, the nature of glycosidic linkages, and more details [27]. The methylation product of TMP-2a’s total ion flow spectrum primarily consists of eight peaks, suggesting that TMP-2a contains eight Polymethacrylic acid sodium (PMAAs) (Figure 4). The mass spectral fragmentation data presented in Table 2 indicate that TMP-2a had eight sugar residues. The finding of TMP-2a’s maximal percentage of glucose content closely matched the findings of the monosaccharide component analysis and served as confirmation of the methylation experiment. Thus, the predominant component in the structure of TMP-2a, →6-Glc*p*-(1→, was the sugar residue present in the greatest abundance.

### 3.7. The NMR Analysis of TMP-2a

**Figure 5:**
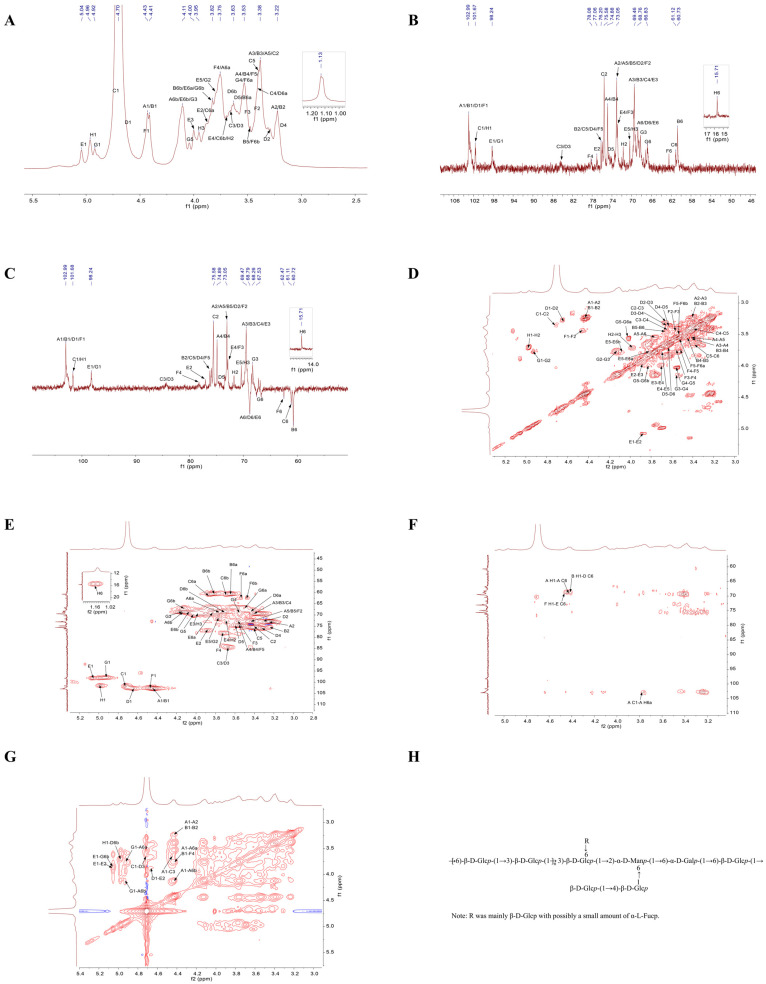
^1^H NMR spectrum (**A**); ^13^C NMR spectrum (**B**); DEPT 135 NMR spectrum (**C**); COSY spectrum (**D**); HSQC spectrum (**E**); HMBC spectrum (**F**); NOESY spectrum (**G**); predicted repeating unit of TMP-2a (**H**).

The structural characteristics of polysaccharides are elucidated by 1D NMR, including ^1^H NMR and ^13^C NMR, as well as 2D NMR techniques such as COSY, HSQC, HMBC, and NOESY [28]. The 1D NMR primarily focused on the arrangement of glycosidic linkages inside polysaccharide structures [29]. Figure 5A illustrates that the hydrogen spectrum signatures of TMP-2a were predominantly located within the range of δ 3.2 to 5.5 ppm. In the anomeric signal area, there were linked peaks from δ 4.4 to 5.8 ppm with chemical shifts of δ 4.43, 4.41, 4.72, 4.64, 5.05, 4.46, 4.92, and 4.97 ppm. These were identified as glycan residues A1, B1, C1, D1, E1, F1, G1, and H1, respectively. The signal at δ 1.2 to 1.4 ppm was frequently regarded as the hydrogen signal of 6-deoxysugar [30], which, in conjunction with the methylation data, designated the site as the signal of fucose H6. The δ 3.2 to 4.2 ppm range contained the majority of the non-anomeric hydrogen signatures. Due to the substantial overlap of the different signals, the H2-H6 chemical shift in each sugar residue needs to be determined separately by integrating the COSY and HSQC spectra.

Compared to ^1^H NMR, the spread of polysaccharide chemical shift signals in ^13^C NMR was broader. The multiple signal peaks were discerned by TMP-2a in this area, with the cross-peaks in the anomeric area of the ^13^C NMR spectra (Figure 5B) and HSQC spectra (Figure 5E) delineating the anomeric signals of residues A, B, C, D, E, F, G, and H as δ 4.43/102.99, 4.41/102.99, 4.72/101.67, 4.64/102.65, 5.05/98.24, 4.46/102.95, 4.92/98.39, and 4.97/101.58 ppm. It was commonly believed that the signal at δ 15.71 ppm was that of fucose C6. Through the integration of the monosaccharide composition results, the methylation analysis findings, anomeric signals, and extensive literature [31,32,33,34], the glycosyl residues of A-H were determined as presented in Table 3. By integrating the ^1^H NMR, COSY, HSQC, and ^13^C NMR spectra, the chemical shifts in each residue were determined (Table 3). The following was the procedure used to attribute the NMR signals to the main sugar residues:

Sugar residue A: Based on the anomeric signal δ 4.43/102.99 ppm (C1/H1), residue A was identified as a β-configured glucose residue. The COSY plot cross-peak δ 4.43/3.24 ppm for residue A (3.24 ppm) identified the H1 chemical shift δ 4.43 ppm of sugar residue A as discovered by ^1^H NMR (Figure 5D). Utilizing the same methodology, the H3 to H6 signals were progressively identified through COSY mapping, with the chemical shifts in sugar residue A assigned as follows: H3 at δ 3.4 ppm, H4 at δ 3.56 ppm, H5 at δ 3.43 ppm, and H6a/(H6b) at δ 3.77 ppm (4.13 ppm), respectively. The chemical shifts for C2 through C6 were identified by HSQC signals as δ 72.99 ppm, δ 69.47 ppm, δ 74.88 ppm, δ 73.05 ppm, and δ 68.76 ppm, respectively, after the hydrogen chemical shifts on the sugar ring were assigned. It was feasible to identify the secondary carbon (negative signal), or the carbon at position 6, by contrasting the DEPT-135 spectrum (Figure 5C) with the ^13^C NMR spectrum. Together with the findings of the methylation study and literature reviews, the chemical shifts in C1 and C6 were moved to the lower field, suggesting that the residues were changed at positions O-1 and O-6 of the sugar ring. These data led to the determination that the sugar residue A was →6)-β-D-Glc*p*-(1→).

Sugar residue B: Residue B was also a glucose residue with a β-configuration, according to the anomeric signal δ 4.41/102.99 ppm (C1/H1). Utilizing the H1 chemical shift δ 4.41 ppm of sugar residue B, ascertained using ^1^H NMR, the H2 to H6 signals were sequentially identified through the cross-peaks of the COSY spectrum, employing the same methodology as for sugar residue A. The H2 to H6 chemical shifts in sugar residue B were assigned to δ 3.24 ppm, δ 3.4 ppm, δ 3.56 ppm, δ 3.46 ppm, and δ 3.63 ppm (3.82 ppm), respectively. The chemical transformations of the sugar ring from C2 to C6 were discerned using HSQC signals at δ 75.81 ppm, δ 69.47 ppm, δ 74.81 ppm, δ 72.97 ppm, and δ 60.71 ppm. The residue was substituted at the O-1 position of the sugar ring, as shown by the chemical shifts in C1, which were moved to the lower field. It was determined that the sugar residue B was β-D-Glc*p*-(1→).

Sugar residue C: In the β-configuration, residue C was likewise a glucose residue, according to the anomeric signal δ 4.72/101.67 ppm (C1/H1). Utilizing the H1 chemical shift δ 4.72 ppm of sugar residue C as determined by ^1^H NMR, the H2 to H6 signals were sequentially identified through the cross-peaks in the COSY spectrum. The chemical shifts for H2 to H6 of sugar residue C were assigned as δ 3.37 ppm, δ 3.66 ppm, δ 3.42 ppm, δ 3.39 ppm, and δ 3.88 ppm (3.69 ppm), respectively. In HSQC, the chemical shifts from C2 to C6 on the sugar ring are indicated by signal imputations of δ 75.58 ppm, δ 84.45 ppm, δ 69.64 ppm, δ 75.91 ppm, and δ 61.12 ppm. When the residues were substituted at the O-1 and O-3 positions of the sugar ring, the chemical shifts in C1 and C3 were shifted to the lower field. The calculated value of the sugar residue C was →3)-β-D-Glc*p*-(1→).

Sugar residue D: In the β-configuration, residue D was likewise a glucose residue, according to the anomeric signal δ 4.64/102.65 ppm (C1/H1). Utilizing the H1 chemical shift δ 4.64 ppm of sugar residue D as determined by ^1^H NMR, the H2 to H6 signals were sequentially identified through the cross-peaks in the COSY spectrum. The chemical shifts for H2 to H6 of sugar residue D were assigned as δ 3.29 ppm, δ 3.67 ppm, δ 3.33 ppm, δ 3.63 ppm, and δ 3.43 ppm (3.72 ppm), respectively. The HSQC signals of δ 73.01 ppm, δ 84.33 ppm, δ 75.81 ppm, δ 74.49 ppm, and δ 68.58 ppm could be responsible for the chemical changes in C2 to C6 on this sugar ring. The residue was substituted at the sugar ring’s O-1, O-3, and O-6 positions, which caused the chemical shifts for C1, C3, and C6 to shift downfield. The sugar residue D was determined to be →3,6)-β-D-Glc*p*-(1→).

Sugar residue E: An α-configuration mannose residue was identified by the anomeric signal δ 5.05/98.24 ppm (C1/H1). Utilizing the H1 chemical shift δ 5.05 ppm of sugar residue E as determined by ^1^H NMR, the H2 through H6 signals were sequentially identified through the cross-peaks in the COSY spectrum. The chemical shifts for H2 to H6 of sugar residue E were assigned as δ 3.86 ppm, δ 3.98 ppm, δ 3.7 ppm, δ 3.78 ppm, and δ 3.83 ppm (4.08 ppm), respectively. The signals δ 77.06 ppm, δ 69.35 ppm, δ 72.58 ppm, δ 70.44 ppm, and δ 69.04 ppm, as indicated in HSQC, may be the cause of the chemical changes from C2 to C6 on this sugar ring. After moving the C1, C2, and C6 chemical shifts to the lower field and replacing the residues at the sugar ring’s O-1, O-2, and O-6 locations, it was determined that the sugar residue E was →2,6)-α-D-Man*p*-(1→).

Sugar residue F: The anomeric signal δ 4.46/102.95 ppm (C1/H1) showed that, in the β-configuration, residue F was a glucose residue. Utilizing the H1 chemical shift δ 4.46 ppm of sugar residue F as determined by ^1^H NMR, the H2 through H6 signals were sequentially identified through the cross-peaks in the COSY spectrum. The chemical shifts for H2 to H6 of sugar residue F were assigned as δ 3.44 ppm, δ 3.57 ppm, δ 3.73 ppm, δ 3.52 ppm, and δ 3.54 ppm (3.48 ppm), respectively. On this sugar ring, the HSQC signals δ 73.17 ppm, δ 72.85 ppm, δ 78.08 ppm, δ 76.36 ppm, and δ 62.47 ppm might be used to identify the chemical changes from C2 to C6. The residues were replaced at the O-1 and O-4 locations of the sugar ring, resulting in a downfield shift in the chemical shifts for C1 and C4. The sugar residue F was determined to be →4)-β-D-Glc*p*-(1→.

Sugar residue G: Residue F was a glucose residue with an α-configuration, according to the anomeric signal δ 4.46/102.95 ppm (C1/H1). Utilizing the H1 chemical shift δ 4.46 ppm of sugar residue G as determined by ^1^H NMR, the H2 to H6 signals were sequentially identified through the cross-peaks in the COSY spectrum. The chemical shifts for H2 to H6 of sugar residue G were assigned as δ 3.78 ppm, δ 4.12 ppm, δ 3.55 ppm, δ 4.02 ppm, and δ 3.52 ppm (3.83 ppm), respectively. This sugar ring’s chemical changes from C2 to C6 were indicated by the HSQC at δ 71.53 ppm, δ 68.35 ppm, δ 69.58 ppm, δ 70.29 ppm, and δ 66.65 ppm. The substitution of the residue at the O-1 and O-6 positions of the sugar ring shifted the chemical shifts in C1 and C6 to the lower field. The sugar residue G was identified as →6)-α-D-Gal*p*-(1→.

Sugar residue H: In the β-configuration, residue F was a sugar residue, according to the anomeric signal δ 4.97/101.58 ppm (C1/H1). ^1^H NMR was used to detect the H1 chemical shift (δ 4.46 ppm) of the sugar residue H, the cross-peak δ 4.97/3.7 ppm of the COSY spectrum indicated the H2 of residue H (3.7 ppm), and the cross-peak δ 3.7/3.96 ppm indicated the H3 of residue H (3.96 ppm). The chemical shifts attributed to C2 and C3 on this sugar ring by HSQC signals were δ 71.84 ppm and δ 70.6 ppm. The positive peak signal at δ 15.71 ppm in the DEPT-135 spectrum signified a methyl peak, which, in conjunction with the methylation result, was ascribed to C-6 of fucose. The cross-peak at δ 1.13/15.71 ppm in the HSQC spectrum represented a characteristic signal of a 6-deoxy sugar. It was determined that the sugar residue H was α-L-Fuc*p*-(1→). Due to the low content of residue H, part of the signal was weakly unidentified (H-4 and H-5).

Based on the chemical shifts in each sugar residue ^13^C and ^1^H, the main linkage modes of the presence of structural fragments and in this polysaccharide were analyzed in combination with the HMBC spectrum (Figure 5F) and the NOESY profile (Figure 5G). The HMBC spectrum showed the cross-peaks of sugar residue A-H1 with residue A-C6 (δ 4.43/68.76 ppm) and A-C1 with residue A-H6a (δ 102.99/3.77 ppm). A cross-peak δ 4.41/68.58 ppm was observed between residue D-C6 and sugar residue B-H1. The sugar residue F-H1 had a cross-peak δ 4.46/69.04 ppm with residue E-C6. This indicated that at least A1→A6, B1→D6, and F1→E6 linkage fragments existed in this polysaccharide. NOESY spectroscopy validated the connectivity sequence of the residues in this polysaccharide, demonstrating cross-peaks at δ 4.43/3.77 ppm for sugar residues A-H1 and A-H6, and δ 4.43/3.66 ppm for sugar residues A-H1 and C-H3. The sugar residue B-H1 exhibited a cross-peak at δ 4.41/3.73 ppm with residue F-H4. At δ 4.72/3.67 ppm, the sugar residue C-H1 and residue D-H3 crossed. It was discovered that the sugar residue D-H1 and residue E-H2 had a cross-peak δ 4.64/3.86 ppm. Alongside residue G-H6, the sugar residue E-H1 showed up with a cross-peak δ 5.05/3.83 ppm. In comparison to residue A-H6, the sugar residue G-H1 displayed a cross-peak δ 4.92/4.13 ppm. At δ 4.97/3.72 ppm, the sugar residue H-H1 and residue D-H6 crossed. Consequently, the integration of monosaccharide composition analysis, methylation, and NMR findings indicated that the primary structure of TMP-2a comprises Glc as the principal chain. It is mainly formed by →6)-β-Glc*p*-(1→ and →3)-β-D-Glc*p*-(1→ to form the main chain with a small amount of →2,6)-α-D-Man*p*-(1→ and →6)-α-D-Gal*p*-(1→ structural units, and the branched chain mainly consists of β-Glc*p*-(1→ or a small amount of α-L-Fuc*p*-(1→ attached as a telosugar to →3,6)-β-D-Glc*p*-(1→ at the O-6 position. There was a portion of β-Glc*p*-(1→ linked to →4)-β-D-Glc*p*-(1→ as a branched chain attached at the O-6 position of →2,6)-α-D-Man*p*-(1→. Thus, the possible structural units of TMP-2a could be surmised based on the available findings (Figure 5H). This study’s AFM results examined the presence of branched chains in TMP-2a polysaccharides, aligning with the findings of the proposed structural analysis.

Glucans are multiple structurally distinct d-glucose polymers, categorized as α-D-glucan, β-D-glucan, and mixed α, β-D-glucans, based on the isomeric structure of glucose [35,36]. Consequently, this structural study indicated that TMP-2a was a β-D-glucan. β-D-glucan was also the most studied active component of fungal polysaccharides with good bioactivity, widely distributed in a large number of edible and medicinal fungi, such as *Pleurotus tuber-regium* [37] and *Laetiporus sulphureus* [38], from which β-D-glucan was also extracted. Previous studies have shown that TMP has (1-4)-β-glucopyranose as the main chain with a branched chain at position O-6, but there were differences in the way the branched chains were connected [10]. The reason for the differences in comparing the structures of TMP might be due to the inconsistencies in the origin of *T. matsutake*, extraction techniques, and the selection of ion chromatography and gel chromatography columns for separation and purification.

### 3.8. In Vitro Immune Activity of TMP

**Figure 6:**
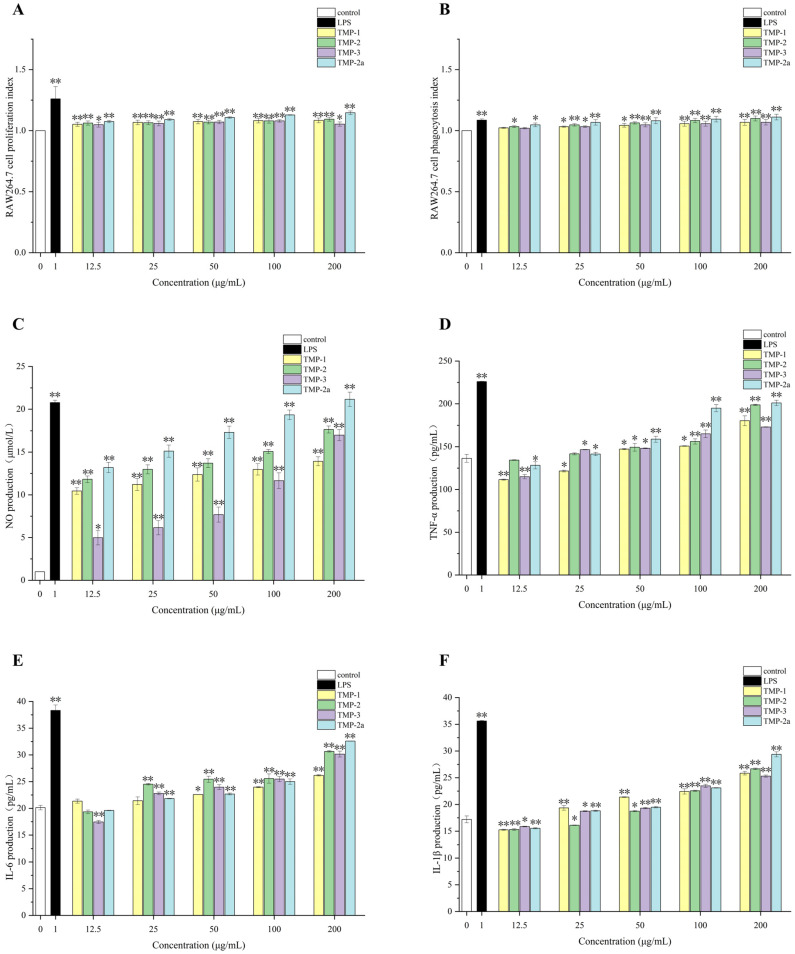
Effect of polysaccharide components on the survival rate of RAW264.7 cells (**A**); effect of polysaccharide components on phagocytic index of RAW264.7 cells (**B**); effects of polysaccharide fractions on NO (**C**), TNF-α (**D**), IL-6 (**E**), and IL-1β (**F**) production in RAW264.7 cells. Data presented the mean ± SD, *n* = 3. Compared with the control group, * *p* < 0.05 and ** *p* < 0.01.

Numerous palatable fungal polysaccharides can efficiently enhance macrophages’ capacity to phagocytose, such as Bulgaria inquinans [39], Lentinus squarrosulus [40], etc. Li et al. [41] identified two water-soluble polysaccharides, RVP-1 and RVP-2, derived from *Russula virescens*. These two polysaccharides may suppress the proliferation of HepG-2, A549, and MCF-7 cancer cells and activate RAW264.7 cells to release immune cytokines, hence mediating the potential for a cellular immune response. Figure 6A illustrates that the multiplication rate of RAW264.7 cells increased as the concentration of TMP decreased. This difference was very significant (*p* < 0.01) in comparison to the blank group. The value-added rate of TMP-3 diminished within the concentration range of 100–200 μg/mL; however, it remained superior to that of the blank control group. This reduction may be attributed to the elevated concentration of polysaccharide, which exerted an inhibitory influence on the proliferative activity of macrophage RAW264.7, aligning with the findings of Gu et al. [42] and Zhu et al. [43]. The proliferative activity of macrophage RAW264.7 began to decrease when the polysaccharide concentration was high. It could be seen that TMP was non-toxic and had a proliferative effect on RAW264.7 cells. Research had indicated that macrophages could be activated by polysaccharides, which could enhance quantity and phagocytic capacity [44].

The phagocytosis performed by macrophages is a crucial component of the immune response [45]. Figure 6B illustrates that LPS in the positive control group markedly enhanced the phagocytosis of RAW264.7 cells relative to the blank control group (*p* < 0.01). Likewise, treatment with TMP-1, TMP-2, TMP-3, and TMP-2a led to increased macrophage phagocytosis within the concentration range of 12.5–200 μg/mL, demonstrating that TMP exhibited considerable macrophage-stimulating action in the immunological response. The phagocytic activity of TMP-2 and TMP-2a was more effective with increasing concentrations. It might be posited that these four components augment the quantity and activity of RAW264.7 cells, hence enhancing phagocytosis and immunomodulatory function through the amplification of innate immunity.

The synthesis of nitric oxide serves as a quantifiable marker for macrophage activation. Figure 6C illustrates that four TMP fractions markedly enhanced NO secretion from RAW264.7 cells, displaying a strong dose–response relationship relative to the control group. TMP-1, TMP-2, TMP-3, and TMP-2a all enhanced the nitric oxide-releasing capability of RAW264.7 cells. Upon stimulating the cells with varying concentrations of the four fractions—TMP-1, TMP-2, TMP-3, and TMP-2a—NO secretion levels attained 13.92, 17.65, 16.99, and 21.16 μmol/L at a concentration of 200 μg/mL, respectively, in contrast to 20.78 μmol/L in the LPS group, with all the results demonstrating significant differences (*p* < 0.05). The impact of TMP-2a was marginally greater than that of the LPS group, demonstrating superior levels of NO release.

Cytokines released by both immune and non-immune cells serve as intercellular signaling molecules, including TNF-α, IL-6, and IL-1β [46]. Figure 6D–F illustrate that TMP-1, TMP-2, TMP-3, and TMP-2a, administered at different concentrations (12.5–200 μg/mL), enhanced the macrophage secretion of TNF-α, IL-6, and IL-1β relative to the blank control. As the concentration climbed, the secretion gradually increased as well. Notably, at a concentration of 200 μg/mL, TMP-2 and TMP-2a promoted TNF-α secretion from macrophages better than TMP-1, and TMP-3, TMP-2, TMP-3, and TMP-2a promoted IL-6 secretion from macrophages better than TMP-1, and the level of IL-1β secretion from TMP-2a was better than that from TMP-1, TMP-2, and TMP-3. This outcome aligned with the findings of Hou et al. [47]. A novel pure polysaccharide (TMP-A) extracted from *T. matsutake* can significantly enhance lymphocyte activity at doses of 50–200 and 100–400 μg/mL in vitro.

The immunological activity of polysaccharides in macrophages is tightly linked to the composition and molecular weight of their monosaccharide constituents [11]. Studies have shown that heteropolysaccharides with different structures in polysaccharides, especially glucose and galactose, showed immunomodulatory responses [48]. It was reported that heteroglycans containing sugars such as mannose, galactose, and fucose extracted from edible mushrooms, which were immunomodulatory heteroglycans, also included glucose, but in varying molar ratios, such as the polysaccharides extracted from *Lentinus edodes* and *Grifolafrondosa* [49,50]. The monosaccharide fractions of the three polysaccharide fractions of TMP-1, TMP-2, and TMP-3 have been determined in the previous phase of this study. Each sample comprised mannose, galactose, and glucose, exhibiting variations in the molecular weights of the constituents. The elevated immunological activity of the three polysaccharide fractions might be associated with the concentration of these monosaccharides. In addition, the immunological effect of TMP-2a was better than the other polysaccharide fractions, which was related to the purity of the polysaccharide, the content of the monosaccharide composition, and the way the polysaccharide was linked. Glucans, fucoidans, mannans, galactans, and xylans have been reported to be polysaccharides with immunostimulatory activity [51,52,53]. The TMP-2a structure in this study was characterized as a glucan and had good immunological activity. It was shown that glycosidic connections involving (1→3)-β-D-Glc*p* and (1→6)-β-D-Glc*p* might significantly influence the immunological activity of the polysaccharide [54,55]. The enhanced immunostimulatory activity of TMP-2a might be attributed to its (1→3)-β-D-Glc*p* and (1→6)-β-D-Glc*p* glycosidic bonds, corroborating the findings of Mueller et al. [56], which indicated that an increased presence of (1→3)-β-D-Glc*p* glycosidic links correlates with improved immunoreactivity.

## 4. Conclusions

TMP-2a, a novel, homogenous, highly pure polysaccharide, was extracted from *T. matsutake* for this investigation. TMP-2a comprises fucose, galactose, glucose, and mannose in a molar ratio of 8.1:22.6:56.8:12.5, with a molecular weight of 27,749 Da. The TMP-2a polysaccharide was mainly composed of →6)-β-Glc*p*(1→ with →3)-β-D-Glc*p*(1→ forming the main chain, with a small amount of →2,6)-α-D-Man*p*-(1→ and →6)-α-D-Gal*p*-(1→ structural units attached, and the branched chain was mainly composed of β-Glc*p*(1→ or a small amount of β -D-Fuc*p*-(1→ was attached as a telose at the O-6 position of residue D. A segment of β-Glc*p*(1→ was conjugated to →4)-β-D-Glc*p*-(1→ and affixed as a branched chain at the O-6 position of residue E. The in vitro immunological activity of TMP-1, TMP-2, TMP-3, and TMP-2a was studied by macrophage RAW264.7. TMP showed good immunological activity, and the effect on the immunological activity of mouse macrophages RAW264.7 was more significant as the concentration increased. The immunological effects of TMP-2, TMP-3, and TMP-2a were superior to those of TMP-1. In addition to providing a theoretical framework for the development and use of foods and medications related to *T. matsutake*, this study lays the groundwork for future investigations into the mechanisms behind the structure and functional activity of TMP.

## Figures and Tables

**Table 1:** The monosaccharide composition, relative molecular weight, and particle size and ζ-potential determination of TMP-2a.

Item	TMP-2a
Monosaccharide composition (%)	
Fucose	8.1
Galactose	22.6
Glucose	56.8
Mannose	12.5
Relative molecular weight	
Retention time (min)	41.02
Mw (Da)	27,749
Mn (Da)	20,896
Particle size and ζ-potential determination	
Particle size (nm)	97.13 ± 0.64
PdI	0.24 ± 0.03
ζ-potential (mV)	−8.30 ± 0.45

**Table 2:** Methylation results of TMP-2a.

Retention Time	Methylated Sugar	Mass Fragments (*m*/*z*)	Molar Ratio	Type of Linkage
11.926	2,3,4-Me_3_-Fuc*p*	43, 59, 72, 89, 101, 115, 117, 131, 175	0.011	Fuc*p*-(1→
16.277	2,3,4,6-Me_4_-Glc*p*	43, 71, 87, 101, 117, 129, 145, 161, 205	0.203	Glc*p*-(1→
20.908	2,4,6-Me_3_-Glc*p*	43, 87, 99, 101, 117, 129, 161, 173, 233	0.167	→3)-Glc*p*-(1→
21.405	2,3,6-Me_3_-Glc*p*	43, 87, 99, 101, 113, 117, 129, 131, 161, 173, 233	0.047	→4)-Glc*p*-(1→
22.622	2,3,4-Me_3_-Glc*p*	43, 87, 99, 101, 117, 129, 161, 189, 233	0.314	→6)-Glc*p*-(1→
24.307	2,3,4-Me_3_-Gal*p*	43, 87, 99, 101, 117, 129, 161, 189, 233	0.046	→6)-Gal*p*-(1→
27.708	2,4-Me_2_-Glc*p*	43, 87, 117, 129, 159, 189, 233	0.141	→3,6)-Glc*p*-(1→
29.256	3,4–Me_2_-Man*p*	43, 87, 99, 129, 189	0.073	→2,6)-Man*p*-(1→

Note: Me—methyl; Fuc*p*—fucose; Glc*p*—glucose; Gal*p*—galactose; Man*p*—mannose.

**Table 3:** ^13^C and ^1^H signal assignments of TMP-2a.

Code	Glycosyl Residues	Chemical Shifts (ppm)					
		H1/C1	H2/C2	H3/C3	H4/C4	H5/C5	H6a, b/C6
A	→6)-β-D-Glc*p*-(1→	4.43	3.24	3.4	3.56	3.43	3.77, 4.13
		102.99	72.99	69.47	74.88	73.05	68.76
B	β-D-Glc*p*-(1→	4.41	3.24	3.4	3.56	3.46	3.63
		102.99	75.81	69.47	74.81	72.97	60.71
C	→3)-β-D-Glc*p*-(1→	4.72	3.37	3.66	3.42	3.39	3.88
		101.67	75.58	84.45	69.64	75.91	61.12
D	→3,6)-β-D-Glc*p*-(1→	4.64	3.29	3.67	3.33	3.63	3.43
		102.65	73.01	84.33	75.81	74.49	68.58
E	→2,6)-α-D-Man*p*-(1→	5.05	3.86	3.98	3.7	3.78	3.83
		98.24	77.06	69.35	72.58	70.44	69.04
F	→4)-β-D-Glc*p*-(1→	4.46	3.44	3.57	3.73	3.52	3.54
		102.95	73.17	72.85	78.08	76.36	62.47
G	→6)-α-D-Gal*p*-(1→	4.92	3.78	4.12	3.55	4.02	3.52
		98.39	71.53	68.35	69.58	70.29	66.65
H	α-L-Fuc*p*-(1→	4.97	3.7	3.96	ND	ND	1.13
		101.58	71.84	70.6	ND	ND	15.71

Note: ND: abbreviation for “not detected”, indicating that it was not identified.

## Data Availability

The original contributions presented in this study are included in the article; further inquiries can be directed to the corresponding authors.
